# Enterotoxin Gene Distribution and Genotypes of *Bacillus cereus* *sensu lato* Isolated from Cassava Starch

**DOI:** 10.3390/toxins13020131

**Published:** 2021-02-10

**Authors:** Jennifer Sánchez-Chica, Margarita M. Correa, Angel E. Aceves-Diez, Laura M. Castañeda-Sandoval

**Affiliations:** 1 Grupo de Microbiología Molecular, Escuela de Microbiología, Universidad de Antioquia UdeA, Calle 70 No. 52-21, Medellín 050010, Colombia; jennifer.sanchez@udea.edu.co (J.S.-C.); margarita.correao@udea.edu.co (M.M.C.); 2 Research and Development Department, Minkab Laboratories, Av. 18 de Marzo No. 546, Col. La Nogalera, Guadalajara P.O. Box 44470, Jalisco, Mexico; aea@minkab.com

**Keywords:** cassava starch, *Bacillus cereus*, enterotoxin, emetic toxin, toxigenic diversity, genomic heterogeneity

## Abstract

*Bacillus cereus* is a human pathogenic bacterium found in foods with the potential to cause emesis and diarrhea. This study estimated the presence, toxigenic and genomic diversity of *B. cereus s.l.* obtained from cassava starch samples collected in bakeries and powdered food companies in Medellín (Colombia). *Bacillus cereus* *s.l.* was found in 43 of 75 (57%) cassava starch samples and 98 isolates were obtained. The *nheABC*, *hblCDAB*, *cytK2*, *entFM* and *cesB* toxin genes were detected by multiplex PCR and the most frequent operon was *nheABC*, whereas *cesB* gene was not found. Twelve toxigenic profiles were determined by the detection of toxin genes, and the most frequent profiles harbored all enterotoxin genes. A broad genomic diversity was detected according to GTG_5_-PCR fingerprinting results with 76 *B. cereus s.l.* grouped in sixteen clusters and the 22 isolates clustering separately. No relationship was observed between genomic background and toxigenic profiles. In general, the results showed a high genomic and enterotoxigenic diversity in *B. cereus s.l.* found in cassava starch. These results should incentive future studies to understand the distribution of *B. cereus s.l.* isolated on raw materials in comparison with finished products.

## 1. Introduction

*Bacillus cereus sensu lato* (*s.l.*) is a group of facultative anaerobic, spore-forming, Gram-positive and motile rod bacteria ubiquitously distributed in the environment [1]. Eight members of the group are recognized, despite their high degree of genetic similarity: *Bacillus anthracis*, *B. cereus sensu stricto* (*s.s*.), *Bacillus cytotoxicus*, *Bacillus mycoides*, *Bacillus pseudomycoides*, *Bacillus thuringiensis*, *Bacillus toyonensis* and *Bacillus weihenstephanensis* [2]. In addition, new species have been proposed based on molecular analysis [3,4]. Some group species are recognized human pathogens [5]; they are naturally found in soil, which enables the contamination of food products and as their spores can survive high temperatures during the cooking processes, consumption of these products may lead to foodborne infection or intoxication [6].

Toxins produced by *B. cereus* cause two types of foodborne diseases in humans, the emetic and diarrheal syndromes [7]. The cereulide toxin causes emesis and it is produced by a non-ribosomal peptide synthetase encoded by the cereulide synthetase (*ces*) gene cluster, located on a megaplasmid [8]. Cereulide is active over a broad pH range (2 to 11), highly heat-stable (121 °C for 90 min) and is produced by *B. cereus* during the stationary phase of growth and under prolonged contaminated food storage [9]. This toxin triggers vacuole formation in cultured HEp-2 and HeLa cells and causes swelling of pig spermatozoa heads, impeding motility [9]. The diarrheal syndrome is produced after colonization of the small intestine by *B. cereus*. At this site, one or more enterotoxins, as the hemolysin BL (HBL), non-hemolytic enterotoxin (NHE) and/or cytotoxin K (CytK), induce diarrhea by stimulating the cAMP system and forming pores in the membrane of epithelial cells [9]. HBL is formed by two lytic proteins, L2 and L1, and a binding protein B, these proteins are encoded by *hblC, hblD* and *hblA* genes, respectively, which compose the *hblCDA* operon; in addition, some *B. cereus* strains carry the *hblB* gene, considered a pseudogene (*hblCDAB* operon) [7]. NHE is formed by a cytolytic protein, NheA and two binding proteins, NheB and NheC, encoded by *nheA, nheB* and *nheC* genes, respectively (*nheABC* operon) [10]. CytK is encoded by the *cytK* gene which has two variants; *cytK1* encodes the CytK1 most toxic variant, while *cytK2* encodes the less toxic and most common variant [7]. Most *B. cereus* strains involved in food-poisoning outbreaks are potential producers of enterotoxin FM (EntFM), encoded by the *entFM* gene, however, there is no evidence that this enterotoxin causes food-poisoning outbreaks [9].

Studies on the presence, genomic and toxigenic heterogeneity of *B. cereus* isolates provide relevant data on their clinical significance, the discovery of contamination sources and help in tracking isolates along the food chain [6]. Various works around the world have reported a high genomic diversity on *B. cereus s.l.* from foods such as ready-to-eat [11,12] and powdered products [13], milk [6,14] and others [15], as well as a broad distribution of toxin genes in *B. cereus s.l.* isolated from foods. For example, in countries as Belgium [11], Brazil [16], Korea [17] and Thailand [18], most *B. cereus* isolates showed presence of all enterotoxin genes. In Colombia, *B. cereus s.l.* isolated from powdered foods [13] and ready-to-eat foods [12] contained the genes for enterotoxins production. Also, a previous study reported the detection of *B. cereus s.l.* toxin genes in DNA extracted directly from cassava starch [19]. Cassava is a tuberous edible plant from the American tropics [20], and in Colombia, the starch derived from cassava is frequently used for cooking a variety of foods including biscuits, soups and prepared meats such as sausages. Usually, cassava starch production is handcrafted, during the process this flour is air-dried on plastic tarps placed on the ground [21], which exposes the product to contamination by microorganisms in the soil, one of them is *B. cereus s.l*. Therefore, the objective of this research was to determine the presence and toxigenic and genomic heterogeneity of *B. cereus s.l.* isolated from cassava starch in Medellín (Colombia).

## 2. Results

### 2.1. Detection of B. cereus s.l. on Cassava Starch

A total of 43 of 75 (57%) cassava starch samples were found contaminated by *B. cereus s.l.* These positive samples were collected in 10 bakeries of 12 sampled and in one powdered-foods company (designated A to M, Table 1) of Medellín (Colombia). Each of the sampling sites were selected to cover the north (A, K, L), south (J, H), east (M, G), west (B, E, I) and center (C, D, F) of the city. The cassava starch suppliers differed for each bakery. By biochemical tests, 98 isolates were confirmed as *B. cereus s.l.* All positive cassava starch samples presented *B. cereus s.l.* counts equal or lower than 1 × 10^3^ UFC g^−1^.

### 2.2. Bacillus cereus s.l. Toxigenic Profiles

**Figure 1:**
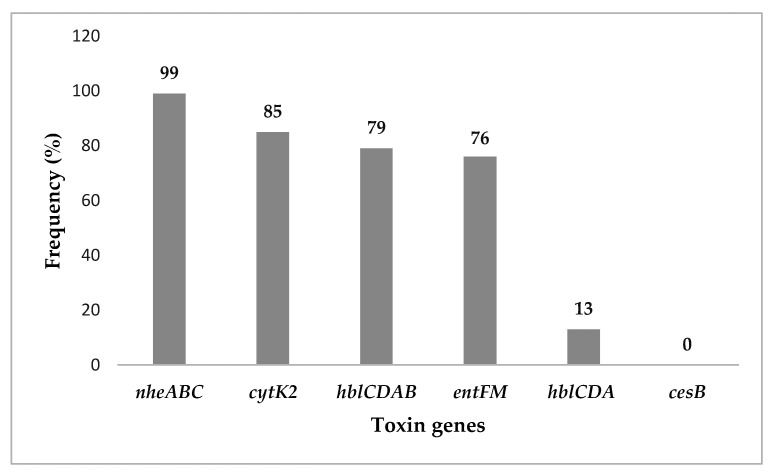
Distribution of toxin genes. Frequency of toxin genes in *B. cereus s.l.* isolated from cassava starch.

Toxigenic determination of the 98 *B. cereus s.l.* from cassava starch by the three multiplex PCR (mPCR) assays showed that 97 isolates harbored all genes of *nheABC* operon (99%), *cytK2* gene was found in 83 isolates (85%), the *hblCDAB* operon in 77 (79%) and *entFM* gene in 74 (76%) isolates. The *hblCDA* operon lacking the *hblB* pseudogene was detected in 13 isolates (13%) and none of the isolates contained the *cesB* gene (Figure 1).

In total, twelve toxigenic profiles were determined according to toxin genes detection (Table 2). Most of the *B. cereus s.l.* (58%) carried profiles with all genes for NHE, HBL, CytK2 and EntFM production (profiles I and II), whereas 21% of the isolates contained the genes for NHE, HBL and CytK2 (profiles III and IV) and 10% of the isolates harbored the genes for NHE, HBL and EntFM (profiles V and VI). Therefore, 58% of *B. cereus s.l.* had the genetic capacity to potentially produce four toxins (profiles I and II), 35% harbored all genes to produce three different toxins (profiles III to VII), 5% had genes for two toxins (profiles VIII to XI) and one isolate had the genes for the NHE enterotoxin exclusively (profile XII).

### 2.3. Genotyping of B. cereus s.l.

**Figure 2:**
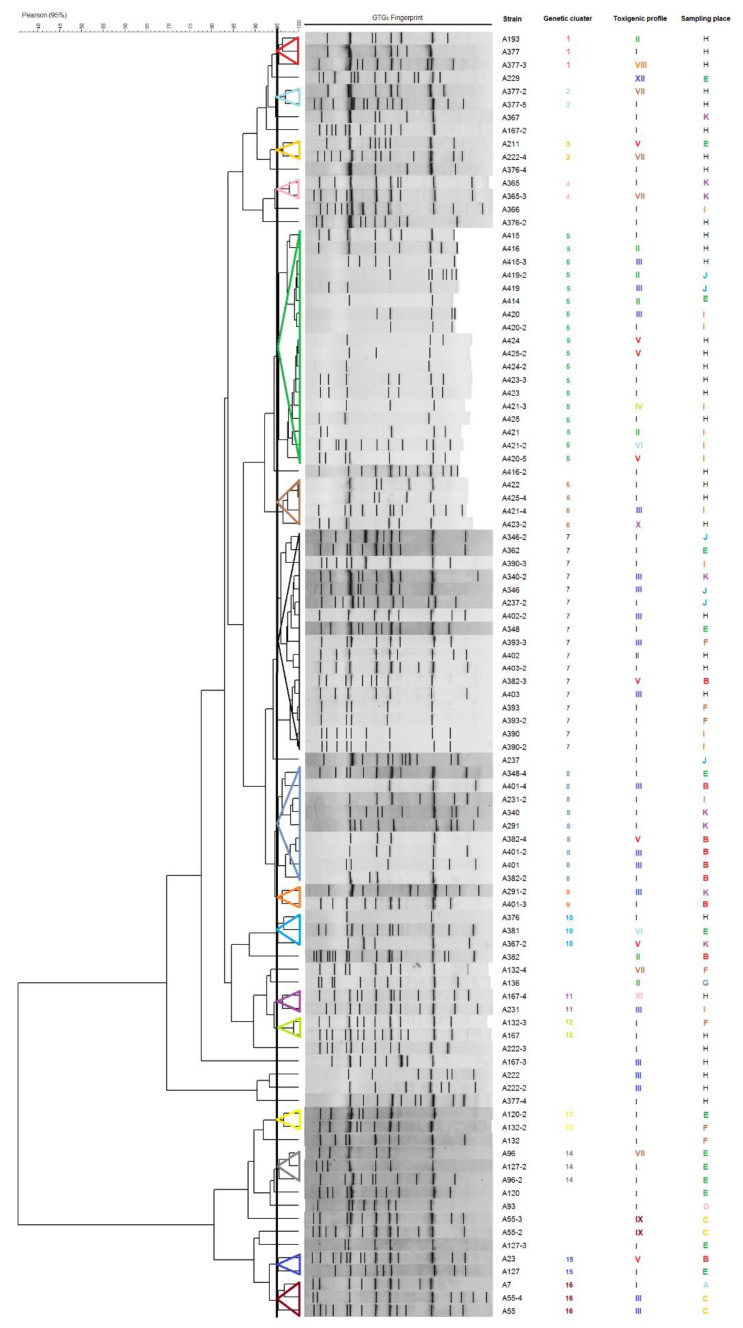
Genotyping of the *B. cereus s.l.* obtained from cassava starch. Dendrogram of (GTG)_5_-PCR fingerprints according to Pearson correlation coefficient (95%) and UPGMA. Colors represent different clusters, toxigenic profiles and sampling places.

**Figure 3:**
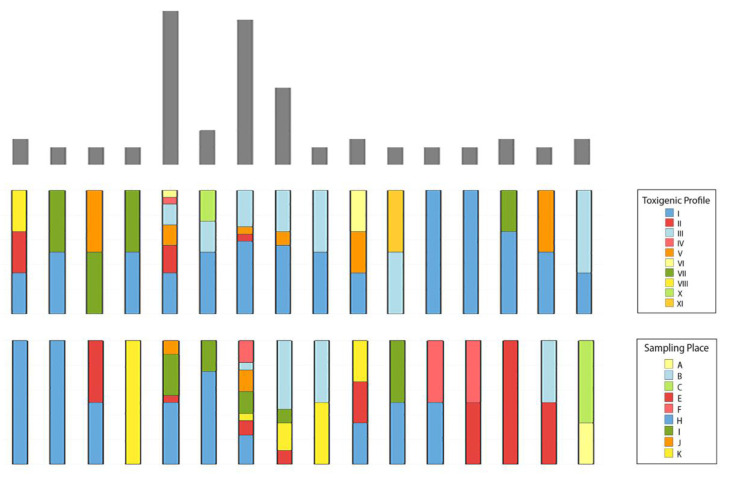
Relationship among genetic cluster, toxigenic profile and sampling place. Each column represents a genetic cluster. The superior bars in grey indicate the number of *B. cereus s.l.* isolates, while the different colors in the bars represent the frequency of toxigenic profile and sampling place, according to the legend.

The genotyping of 98 *B. cereus s.l.* isolated from cassava starch using (GTG)_5_-PCR fingerprinting showed that sixteen clusters were formed by 76 *B. cereus s.l.* isolates and 22 isolates clustered separately (Figure 2). A large diversity was observed on clusters 5, 7 and 8, which were composed by isolates from at less four sampling places and three toxigenic profiles (Figure 2 and Figure 3). In contrast, clusters 1, 2, 4 and 14 each contained isolates from a single sampling place. Interestingly, clusters 12 and 13 contained isolates from different sampling places but had the same toxigenic profile. Regarding toxigenic profiles and genetic clusters, 10 of 12 toxigenic profiles were distributed in the sixteen genetic clusters while toxigenic profiles IX and XII were exclusive of isolates clustered individually.

## 3. Discussion

*Bacillus cereus s.l.* was detected in 43 of 75 (57%) cassava starch samples. This is considered a relatively high incidence if compared to values obtained during the analysis of other food matrices in Colombia such as powdered foods that showed incidences from 10% to 13% [13]. Possibly, the high presence of *B. cereus s.l.* in the cassava starch is a consequence of its handcrafted manufacturing process, in which flour contamination with *B. cereus* occurs during the drying of the flour on plastic tarps placed on the ground under the sun, in an open-air environment. Besides, the basic microbiological analysis of this food matrix which comprises testing for fungi, yeast, total aerobic mesophilic bacteria and *Escherichia coli*, it does not include detecting *B. cereus s.l.,* which impedes a better control of this pathogenic bacterium in the cassava starch [22]. Worldwide, incidences of *B. cereus s.l.* are variable; the values reported are either higher or lower than the ones obtained in this study; however, as long as the food raw-materials and flours are handcrafted and slightly processed, they are susceptible to contamination by *B. cereus* spores and vegetative cells, constituting great risk for human health [12,13]. Studies performed in countries such as Korea found *B. cereus* in 40% of raw rice samples [23]; in Iran, 42% of infant formula analyzed carried *B. cereus* [24] and in New Zealand, *B. cereus* incidences in dehydrated potato ranged from 10% to 40% [25]. In Belgium, variable incidences of *B. cereus s.l.* (5% to 100%) were informed in ready-to-eat foods [11], and, in Scotland, reports of this pathogen varied from 20% to 88% in soil, feces and vegetables samples [26]. In this work, all positive cassava starch samples presented *B. cereus s.l.* counts equal or lower than 1 × 10^3^ UFC g^−1^, and although, these amounts are considered safe for human consumption [27], similar *B. cereus* counts have been found in foods causing disease [7]. Considering that eleven of thirteen sampling places presented cassava starch samples contaminated by *B. cereus s.l.*, it is recommended to maintain high baking temperatures for products using cassava starch as the raw material, this with the aim of eliminating *B. cereus s.l.* spores and vegetative cells in the finished product.

The enterotoxin genes showed a broad distribution in the *B. cereus s.l.* isolated from cassava starch. Mainly, the *nheABC* operon was detected among the isolates (99%), carrying all three genes for the non-hemolytic enterotoxin production. This finding is common to those from studies in Brazil [16], Korea [28] and Thailand [18], which have informed that *B. cereus* isolated from foods and soil harbored all genes of the *nheABC* operon. Furthermore, bioinformatic analyses suggested that the *nheABC* operon is highly conserved in *B. cereus s.l.* strains, mainly because of the vertical inheritance; but it is not clear yet if this operon has an additional biological function [29]. The *cytK2* gene was detected in 85% of *B. cereus s.l*. Similar frequencies of this gene were observed for *B. cereus s.l.* strains isolated from powdered foods in Colombia, with a 62% occurrence of *cytK2* [13]. The *B. cereus s.l.* isolated from cassava starch samples showed a high frequency of *cytK2* gene, but the frequencies generally reported (45% to 89%) [11,16,18,30], denote that *cytK2* is broadly distributed among *B. cereus s.l.*

The *hblCDA* operon was found in 90 *B. cereus s.l.* (92%), from which 77 isolates (85%) harbored the *hblB* pseudogene. These isolates with *hblCDA* operon carry all genes that should encode an active hemolysin BL. Some studies report that *B. cereus s.l.* isolates from foods may present polymorphisms in the *hblCDA* operon, as reflected by the lack of one or two operon components [13,17,31]. The frequencies on *B. cereus* carrying *hblCDA* operon genes highly vary among countries and samples evaluated. Frequencies as high as 84% to 100% have been reported in studies performed in Belgium [11] and Korea [28]; whereas lower frequencies (50% to 70%) were reported in Denmark [32] and Brazil [16]. Furthermore, in this study, the *entFM* gene was detected in 74 isolates (76%). This result agrees with previous studies showing high frequencies of *entFM* gene in *B. cereus s.l.* [17,24,33,34].

None of the 98 *B. cereus s.l.* isolated from cassava starch harbored the *cesB* gene. The no detection of presumptive emetic strains in this study is probably due to the low frequency of these strains that belong to a special lineage of *B. cereus s.s*. [35]. It is suggested that the use Bacara agar could improve the isolation of emetic *B. cereus* strains given that this medium is more selective than MYP because it impedes the growth of other Gram-positive microorganisms commonly found in foods [36]; therefore, future studies should incorporate this agar to see if the detection of emetic strains is improved. These emetic strains contain the megaplasmid that harbors the cereulide synthetase (*ces*) gene cluster [35] and according to biochemical and molecular test, share various phenotypic and genotypic characteristics that include, hydrolysis of starch and salicin, haemolytic activity, and genotypic/protein patterns in assays such as random amplification of polymorphic DNA (RAPD), multilocus sequence typing (MLST), phenetic Fourier transform Infrared (FTIR) and protein profiling [37]. Studies in countries such as Belgium [11], Brazil [16] and China [38] have reported low frequency of emetic strains, or they are even not detected. Meanwhile, in Asian countries, e.g., Korea, *B. cereus* emetic strains have been detected in rice [28]. In general, the finding of emetic strains mainly in oriental rice dishes could indicate a relationship between *B. cereus* emetic strains and rice [5].

According to the detection of the toxin genes *nheABC*, *hblCDAB*, *cytK2*, *entFM* and *cesB*, twelve different toxigenic profiles were identified. The majority of the *B. cereus s.l.* isolates (58%) carried profiles with all genes for NHE, HBL, CytK2 and EntFM production (profiles I and II). This result resembles those obtained in other countries such as Belgium [11], Brazil [16], Korea [17] and Thailand [18], where the most frequent profiles harbored all enterotoxin genes in *B. cereus* isolated from foods. In addition, in a previous study performed in Colombia that evaluated the toxin genes of *B. cereus s.l.* isolated from flours and powdered milk, found that the most frequent toxigenic profile included all enterotoxin genes. Altogether, the results reinforce the high degree of conservation of enterotoxin genes in *B. cereus s.l.* strains isolated from powdered food and other matrices [13]. Furthermore, the data suggest that the *B. cereus s.l.* isolated from cassava starch are potentially able to cause just the diarrheal syndrome, but further studies directed to detect the levels and simultaneity of expression of all toxins will indicate if they have the potential to cause foodborne illness.

Genotyping by (GTG)_5_-PCR fingerprinting of the *B. cereus s.l.* isolated from cassava starch showed a high diversity. According to sampling places or toxigenic profiles, the respective isolates get distributed on various clusters. In general, no association was found between clusters and collection places; however, clusters 1, 2, 4 and 14, each contained isolates from a single sampling place, which may indicate particular contaminant strains in these places. In addition, clusters 12 and 13 exclusively contained isolates of toxigenic profile I, but from various collection places (E, F and H) and cassava starch suppliers. All toxigenic profiles, except IX and XII, were spread among the different clusters, whereby, *B. cereus s.l.* isolates carrying the same toxigenic profile presented different banding patterns and all clusters, except clusters 12 and 13, presented different toxigenic profiles. These results suggest that there is no relationship between genetic background and toxigenic profiles, as previously reported [13,15,16].

In general, the results of this study indicate that *B. cereus s.l.* isolates obtained from cassava starch have the potential to cause diarrheal-type foodborne illness; this given the fact that the isolates harbor the genes codifying for the HBL, NHE and CytK2 toxins. The *B. cereus s.l.* genotyping revealed a high genomic heterogeneity among the isolates; this and the high number of toxigenic profiles detected, suggest the presence of different species of *B. cereus* group, as *Bacillus cytotoxicus*, *Bacillus mycoides*, *Bacillus pseudomycoides*, *Bacillus thuringiensis*, and *Bacillus weihenstephanensis*, among the isolates, whereby further studies are needed to confirm it and to characterize the species. This study provides useful information for the assessment of the potential risk of the cassava starch samples containing this pathogen to cause foodborne illness; in addition, it could help to a better comprehension of the epidemiology of *B. cereus s.l.* in this raw material. Finally, these results should incentive additional studies to understand the distribution of *B. cereus s.l.* on raw materials as cassava starch, compared to finished food products and to encourage health politics for the microbiological control of *B. cereus s.l.* in raw materials used for manufacturing traditional Colombian foods.

## 4. Materials and Methods

### 4.1. Cassava Starch Samples and B. cereus s.l. Isolation

A total of 75 cassava starch samples were randomly collected in twelve bakeries and one powdered-foods company of Medellín (Colombia). Isolation of *B. cereus s.l.* from cassava starch was done according to the ISO 7932:2004 norm for the enumeration of presumptive *B. cereus* cells [27]. Concisely, 225 mL of peptone water was used to dissolve 25 g of cassava starch and serial dilutions were carried out until 10^−3^, and 100 μL of each dilution was spread on mannitol egg yolk polymyxin agar (MYP) (Merck Millipore, Darmstadt, Germany); and the MYP plates were incubated at 37 °C for at least 48 h. According to the norm, five presumptive colonies of each cassava starch sample were analyzed using biochemical tests following standard protocols [27]. Biochemical test included catalase, motility, Voges-Proskauer reaction, glucose, xylose and arabinose utilization, β-hemolytic activity and, starch, casein and gelatin hydrolysis. The confirmed *B. cereus s.l.* isolates were conserved in Tryptic Soy Broth and 15% (*v*/*v*) glycerol and stored at −80 °C. Some reference strains (*B. cereus* NVH 1257, F4810/72, ATCC 14579 and ATCC 10987) served as positive controls for biochemical test and molecular analysis.

### 4.2. B. cereus s.l. Toxin Genes Detection

For DNA extraction, *B. cereus s.l.* isolates and *B. cereus* reference strains were grown in Luria-Bertani (LB) broth (Difco, Detroit, MI, USA), incubated overnight at 37 °C. DNA extraction was performed according to previously described methodology [39]. The detection of *nheABC*, *hblCDAB*, *cytK2*, *entFM* and *cesB* toxin genes was done by three multiplex PCR assays as previously described [12].

### 4.3. Bacillus cereus s.l. (GTG)_5_-PCR Fingerprinting

The genomic heterogeneity of *B. cereus s.l.* isolated from cassava starch was evaluated by repetitive element palindromic-polymerase chain reaction (rep-PCR) using (GTG)_5_ primer, as described previously [13]. Briefly, the 20 μL reaction contained 0.6 μM of (GTG)_5_ primer, 200 μM of dNTPs mix, 3.5 mM of MgCl_2_, 1.6 U of Taq DNA polymerase (Thermo Fisher Scientific, Waltham, MA, USA ), 2 μL of 10X PCR buffer and 50 ng of DNA template. PCR consisted of, an initial denaturation of 5 min at 94 °C, followed by 30 cycles of denaturation at 94 °C for 1 min, annealing at 45 °C for 1 min and elongation at 65 °C for 8 min, and a final extension at 65 °C for 16 min. PCR products were electrophoresed in a multiSUB Choice electrophoresis system (Cleaver Scientific, Warwickshire, UK), with a 15 × 15 cm gel tray, 1.5% (*w*/*v*) agarose gel (Amresco, Solon, OH, USA), with ethidium bromide for 2.5 h, at a constant voltage of 120 V in 1X TBE buffer, at 4 °C. The gene ruler 1 Kb DNA ladder (Thermo Fisher Scientific, Waltham, MA, USA) was used as a size standard. The resulting fingerprints were analyzed using the BioNumerics 6.6 software package (Applied Maths Inc., St Martems, Belgium), with 1% optimization and 1% position tolerance. The Pearson correlation coefficient (95%) and the unweighted pair group method with arithmetic average (UPGMA) were used to obtain a dendrogram of the profiles for cluster analysis.

## Figures and Tables

**Table 1:** Distribution of *B. cereus s.l.* obtained from cassava starch according to the sampling place.

Place	Type	Collected Samples (*n*)	Positive Samples (*n*)	*B. cereus s.l.* (*n*)
A	Bakery	3	1	1
B	Bakery	4	3	9
C	Bakery	1	1	4
D	Bakery	1	1	1
E	Bakery	14	9	14
F	Bakery	9	2	7
G	Bakery	1	1	1
H	Powdered foods company	14	13	34
I	Bakery	10	5	13
J	Bakery	11	3	6
K	Bakery	5	4	8
L	Bakery	1	0	0
M	Bakery	1	0	0
TOTAL		75	43	98

**Table 2:** Toxigenic profiles of 98 *B. cereus s.l.* obtained from cassava starch.

Profile	Toxin Genes	Number of *B. cereus s.l.* (%)
I	*nheABC, hblCDAB, cytK2, entFM*	49 (50)
II	*nheABC, hblCDA, cytK2, entFM*	8 (8)
III	*nheABC, hblCDAB, cytK2*	19 (20)
IV	*nheABC, hblCDA, cytK2*	1 (1)
V	*nheABC, hblCDAB, entFM*	8 (8)
VI	*nheABC, hblCDA, entFM*	2 (2)
VII	*nheABC, cytK2, entFM*	5 (5)
VIII	*nheABC, cytK2*	1 (1)
IX	*nheABC, hblCDA*	2 (2)
X	*nheABC, entFM*	1 (1)
XI	*hblCDAB, entFM*	1 (1)
XII	*nheABC*	1 (1)

## Data Availability

The data presented in this study are available upon request to the corresponding author. The data are not publicly available at this moment because the doctoral candidate Jennifer Sánchez-Chica is in the process of thesis defense. Subsequently, it will be delivered to the public repository of the University of Antioquia, where the data will be publicly available.
